# Natural killer cells in tumor immunotherapy

**DOI:** 10.20892/j.issn.2095-3941.2023.0215

**Published:** 2023-08-26

**Authors:** Dongyao Wang, Haiming Wei

**Affiliations:** 1 Department of Hematology, The First Affiliated Hospital of USTC, Division of Life Sciences and Medicine, University of Science and Technology of China, Hefei 230027, China; 2 Institute of Immunology and the CAS Key Laboratory of Innate Immunity and Chronic Disease, School of Basic Medicine and Medical Center, University of Science and Technology of China, Hefei 230001, China; 3 The CAS Key Laboratory of Innate Immunity and Chronic Disease, School of Basic Medicine and Medical Center, University of Science and Technology of China, Hefei 230027, China; 4 Blood and Cell Therapy Institute, Anhui Provincial Key Laboratory of Blood Research and Applications, University of Science and Technology of China, Hefei 230027, China

Cancer is the second most common cause of death worldwide and remains one of the critical public health problems of our time^1^. Recently, immunotherapy has considerably improved the outcomes of patients with advanced cancers. Immune checkpoint blockade and chimeric antigen receptor (CAR)-T cell-based therapies have achieved remarkable success in recent decades, thus placing the host immune response in the spotlight as a potential new approach in antitumor therapy. However, the overall clinical response rates still require to be increased^2^. Natural killer (NK) cells play an integral role in the immune surveillance of cancers and in defense mechanisms against microbial infections^3^. NK cells can potentially eliminate tumor cells *via* receptor-ligand interactions, by releasing cytotoxic granules containing perforin and granzyme, through death receptor-mediated pathways, and secreting a range of effector molecules, such as interferon (IFN)-γ and tumor necrosis factor (TNF)-α^3^. However, in several types of human tumors, the number of NK cells is lower than that in healthy control tissue, and the cells themselves become dysfunctional, thus rendering NK cell-mediated tumor surveillance ineffective^3^. The consequences of these changes and several potential approaches to overcome them are discussed in this editorial. Additionally, we describe recent progress in the genetic engineering and clinical application of CAR-NK cells, briefly discussing the challenges and future promise of these cells in cellular immunotherapy for cancer.

## Immunotherapy targeting NK cells

### Checkpoint targeting to promote NK cell antitumor immunity

NK cell activation is orchestrated by a suite of activating, inhibitory, and co-stimulatory receptors. Cancer cells overexpress ligands for activating receptors, including natural killer group 2 (NKG2), member D ligands, such as UL16-binding proteins, major histocompatibility complex (MHC) class I polypeptide-related sequence A (MICA) and MICB^3^. Downregulation of the expression of these ligands is considered a mechanism of tumor immune evasion. Targeting the MICA α3 proteolytic site promotes NK cell antitumor activity in mice^3^. In addition, NK cells eliminate tumor cells with downregulated expression of MHC molecules known as human leukocyte antigen (HLA) class I molecules^3^. MHC class I molecules bind various inhibitory killer cell immunoglobulin-like receptors (KIRs) and weaken NK cell effector function, thus minimizing the destruction of normal self-cells. Anti-KIR antibodies have been tested alone or in combination with checkpoint therapies in phase I and phase II clinical trials for lymphoid malignancies; however, little clinical efficacy has been documented^4^.

**Figure 1:**
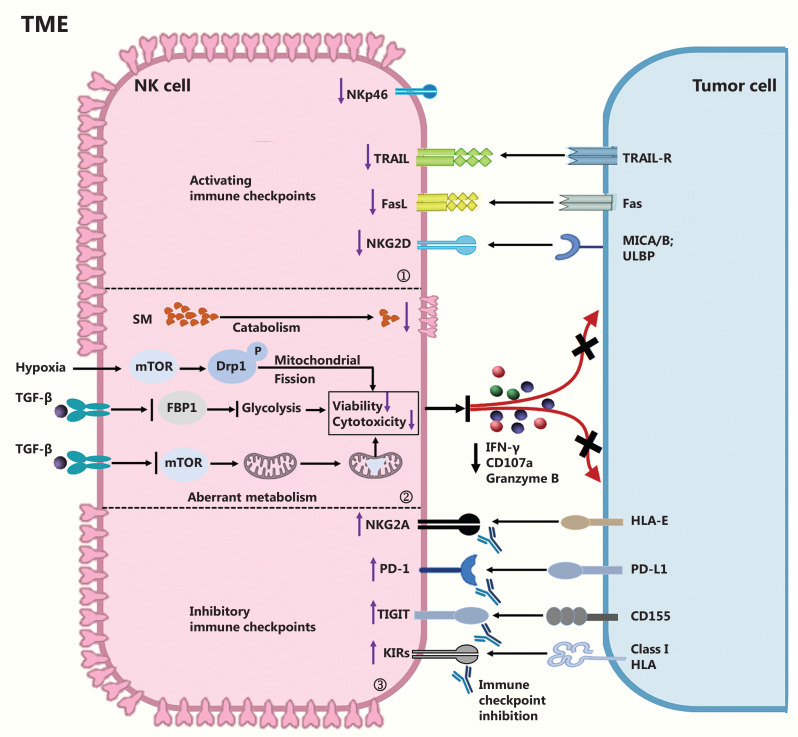
Summary of various approaches to targeting NK cell dysfunction and enhancing NK cell effector function. ① Activating immune checkpoints. Tumor necrosis factor (TNF)-related apoptosis-inducing ligand (TRAIL), Fas ligand (FasL), and NKG2D are activating immune checkpoints expressed on NK cells that can trigger cytotoxicity. Inducing the expression of these molecules and their ligands can restore the antitumor effects of NK cells and make tumor cells more susceptible to immune destruction. ② Targeting aberrant metabolism of NK cells in the tumor microenvironment (TME). TGF-β, FBP1, and a hypoxic TME elicit NK cell dysfunction by impairing mitochondrial homeostasis, metabolism, and viability. Additionally, the sphingomyelin (SM) content and the surface topology of NK cells are often abnormal in the TME. Wei’s group has found that therapeutically targeting TGF-β signaling, FBP1, or sphingomyelinase with blocking monoclonal antibodies or small molecule inhibitors restores NK cell antitumor activity in the immunosuppressive TME. ③ Targeting inhibitory immune checkpoints. Inhibition of NK cell inhibitory receptor signaling with anti-NKG2A (monalizumab), anti-PD-1 (pembrolizumab), anti-TIGIT (tiragolumab), or anti-KIR (lirilumab) enhances NK cell cytotoxicity and the antitumor response.

In the tumor microenvironment (TME), NK cell antitumor responses are progressively impaired by increased ligand binding of the inhibitory NKG2A/CD94 receptor. Targeting NKG2A with the humanized anti-NKG2A antibody monalizumab has been found to enhance NK cell activity against various tumor cells^3^. Additionally, PD-1 is expressed in some NK cells. Interestingly, Hasim^5^ has reported that, rather than endogenously expressing PD-1, NK cells acquire PD-1 from tumor cells *via* trogocytosis. Trogocytosed PD-1 is functional and suppresses the antitumor activity of NK cells. Blocking PD-1/PD-L1 interaction with checkpoint inhibitors enhances NK cell effector function^5^. Furthermore, T cell immunoreceptor with immunoglobulin and ITIM domain (TIGIT), which is upregulated by immune cells including NK cells, is a promising new target for tumor immunotherapy. TIGIT binds the CD155 expressed by tumor cells. Several monoclonal antibodies targeting TIGIT have been developed. Notably, dual PD-1 and TIGIT blockade may be a promising combinatorial immunotherapy for tumors^3,5^ (**Figure 1**).

### Targeting with small-molecule mediators to enhance NK cell antitumor function

In general, developing NK cell-mediated treatments poses 2 main challenges: enhancing the effector function of NK cells while increasing their persistence *in vivo*, and optimizing the sources of NK cells. Wei et al.^6,7^ have revealed that the survival of NK cells is metabolically demanding and that metabolic conditions undergo unfavorable changes in many aberrant tissue microenvironments. Lung cancer is the most common cause of cancer related deaths in both men and women, and is followed by prostate and colorectal cancers in men, and breast and colorectal cancers in women^1^. Wei et al.^6^ have demonstrated that although NK cells prevent lung cancer initiation, they do not control the progression of established tumors. The authors have also found that aberrant fructose-1,6-bisphosphatase (FBP1) expression in tumor-associated NK cells inhibits glycolysis, thereby impairing their survival, promoting transforming growth factor β (TGF-β) production, and leading to lung cancer progression. Furthermore, pharmacologic inhibition of FBP1 restores the antitumor response, thus suggesting that the reprogramming of glucose metabolism might increase NK cell-based cancer therapy^6^.

Liver cancer is rapidly becoming one of the most fatal cancers, and the incidence of these malignancies is growing rapidly^7,8^. Mitochondria are highly dynamic, and their function is closely associated with their morphology, which in turn is influenced by fusion and fission^7^. NK cell survival is metabolically demanding. Fission of mitochondria into fragments accelerates the production of reactive oxygen species, maintains calcium homeostasis, and mediates apoptosis^8^. Wei et al.^7^ have detected highly fragmented mitochondria in the cytoplasm of tumor-infiltrating NK cells in patients with liver cancer. Importantly, this excessive fragmentation of mitochondria appears to correlate with a deficiency in NK cell numbers, decreased NK cell antitumor activity, and predicted poor survival in affected patients. This research group has also demonstrated that the hypoxic TME in liver cancers promotes the constitutive activation of mechanistic target of rapamycin-GTPase dynamin-related protein 1 (mTOR-Drp1) in tumor-infiltrating NK cells, and this change in gene expression induces the fission of mitochondria into fragments. Therefore, hypoxia in liver cancers might be immunosuppressive, driving the escape of tumor cells from NK cell-mediated immunosurveillance^7^. Furthermore, inhibition of mitochondrial fragmentation with the small molecule inhibitor mdivi-1 has been found to increase mitochondrial metabolism, thus benefiting the antitumor function of NK cells *in vivo*^7^.

Immunological synapses play a key role in the progression of cellular cytotoxicity^8^. Previous reports have indicated that membrane protrusions are key components of immune synapses^8,9^. Zheng et al.^9^ have reported that NK cells isolated from liver cancer form fewer membrane protrusions than those derived from healthy tissues or immune cells isolated from peripheral blood. In addition, the authors have demonstrated that dysregulated serine metabolism within the TME leads to a decrease in the sphingomyelin content of NK cells residing in tumors (**Figure 1**). The blockade of sphingomyelin catabolism, through targeting of sphingomyelinase, promotes the antitumor activity of NK cells^9^.

Furthermore, although activation of the stimulator of interferon genes (STING) pathway promotes antitumor immunity, STING agonists have yet to achieve clinical success^10^. Knelson et al.^10^ have reported that STING agonists enhance the migration and killing of NK cells, thereby increasing therapeutic activity in patient-derived organotypic tumor spheroids. By functionally profiling mesothelioma tumor explants with elevated STING expression in cancer cells, the authors have uncovered distinct consequences of STING agonist treatment in humans that support testing of a combination of STING agonists with NK cell therapies.

### NK cell-mediated antibody-dependent cellular cytotoxicity

Antibody-dependent cellular cytotoxicity (ADCC) is a key mechanism of NK cells that is mediated by therapeutic monoclonal antibodies. ADCC is critical for the tumoricidal effects of therapeutic antibodies, in which NK cells bind tumor cells *via* antibodies and lyse them by releasing perforin and granzymes^11^. Li et al.^11^ have reported that monoclonal antibodies, such as trastuzumab and pertuzumab, bind *via* the fragment crystallizable region (Fc) of immunoglobulin G1 to CD16 expressed on NK cells and elicit the release of cytotoxic factors. Therefore, NK cell-mediated ADCC in drugs targeting HER2-positive breast cancer and Fc-optimized anti-HER2 agents would have desirable clinical effects. Understanding the roles of ADCC and the enhancement of NK cell activity could contribute to tumor immunotherapy.

## Targeting the TME to improve the survival and function of NK cells

### Extracellular matrix in the TME

The TME is widely considered to play a crucial role in epigenetic reprogramming, tumorigenesis, and tumor progression, and has been shown to influence immune escape mechanisms^12^. The few NK cells found in solid tumors invariably exhibit abnormal phenotypes and show functional defects^12^. Such NK cell dysfunction has been documented in patients with lung cancer, prostate cancer, breast cancer, hepatocellular carcinoma, and gastrointestinal stromal tumors^12,13^, wherein the frequency of NK cells is markedly lower than that of other myeloid or lymphoid cells. The extracellular matrix (ECM) is an acellular three-dimensional network that forms a scaffold in solid organs^12,13^. Although the ECM limits the initial spread of malignant cells, it also limits the entry of immune cells^12,13^. Proteins belonging to the collagen superfamily account for approximately 30% of the total protein content of the ECM^12,13^. A recent study has demonstrated that a collagen-specific receptor tyrosine kinase, discoidin-domain receptor 1 (DDR1), realigns collagen fibers into a denser structural barrier, thus impeding T-cell infiltration into breast tumors^14^. In the TME, ECM-associated proteins have been implicated in modulating NK cell behavior. NK cells express ECM receptors that regulate both their homeostatic and effector functions^12,13^. For example, collagen-I impairs NK cell cytotoxicity and IFN-γ secretion, thus limiting the effectiveness of cytotoxic NK cell responses in solid tumors^12,15^. Multiple strategies aimed at modifying the protein structure of the ECM are being explored, with the aim of increasing NK cell responses in ECM-rich cancers^12^. For example, simtuzumab, a lysyl oxidase-like 2 (LOXL2)-targeting antibody, has been found to decrease collagen cross-linking and synthesis. Recently, simtuzumab has been tested in the immunotherapy for pancreatic cancer^12^. Altering ECM-NK cell interactions is anticipated to serve as a strategy to promote NK cell-mediated cytotoxicity within the TME.

### Targeting TGF-β1 to improve the survival and function of NK cells

A series of immunosuppressive mechanisms can disable NK cells. For example, TGF-β1 negatively affects NK cell activity^15^. Leukemia is the most common childhood cancer, accounting for 28% of malignancies^1,16^. Wang et al.^15^ have found that bone marrow-derived NK cells isolated from patients with relapsed acute myeloid leukemia after allogeneic hematopoietic stem cell transplantation often show dysfunctional behavior. In addition, activation of TGF-β1, induced by glycoprotein-A repetitions predominant (GARP), impairs mTORC1 activity, mitochondrial oxidative phosphorylation, and the effector function of bone marrow-derived NK cells *ex vivo* (**Figure 1**). Pharmacologic blockade of TGF-β1 signaling with galunisertib contributes to mTOR activation and mitochondrial homeostasis. Importantly, blockade of TGF-β1 signaling has been found to restore NK cell-mediated antileukemic activity in a xenograft mouse model^15^.

## Development of CAR-NK cells

Despite the FDA approval of CD19-targeted CAR-T cell therapy for the treatment of refractory B-cell acute lymphoblastic leukemia and B-cell non-Hodgkin lymphoma, allogeneic CAR-T cells have several limitations^17^. The intervention can result in life-threatening graft-*versus*-host disease (GVHD) and neurotoxicity. Furthermore, when *in vitro* expanded cells are infused into patients, they may suddenly release large amounts of IL-1a, IL-6, TNF-α, or IL-8, thus causing cytokine release syndrome (CRS)^17,18^. In addition, CAR-T cells can be rapidly eliminated by the recipient’s immune system^17,18^. In contrast to T cells, activated NK cells usually produce IFN-γ and granulocyte-macrophage colony-stimulating factor (GM-CSF), thereby limiting the severity of CRS, and their administration is associated with a significantly diminished risk of GVHD^19^. Therefore, the development of CAR-NK cells is a promising alternative for CAR-T therapy.

The development of CAR-NK cells has been fueled by their potential advantages. NK cells can directly recognize certain tumor cells without strict HLA matching. This HLA-independent action, combined with the previously described lack of GVHD and low probability of CRS, suggests that CAR-NK cells could potentially become “off-the-shelf” products^18^. Several ongoing clinical trials registered on clinicaltrials.gov are exploring this possibility. These trials are aimed at evaluating the effectiveness of CAR-NK cell therapy in both hematologic and solid tumors, including prostate cancer, glioblastoma, and ovarian cancer^18^. Currently, most CAR-NK cell therapies target lineage markers on hematopoietic malignancies, such as CD19, CD33, or CD7. Furthermore, CAR-NK cells recognizing solid tumor-associated antigens, including human epidermal growth factor receptor 2 (HER2) and Mucin 1 (MUC1), have also been developed^19^. The results of the first large-scale CAR-NK cell trial have recently been reported. Allogeneic umbilical cord blood-derived CAR-NK cells have been tested in 11 patients with high risk CD19^+^ B-cell malignancies, after standard lymphodepletion. Seven of the 11 patients achieved complete remission, without serious adverse effects, such as CRS or neurotoxicity^18^. Li et al.^20^, using different *in vivo* tumor models and clinical data, have demonstrated that CAR activation in NK cells promotes transfer of the trogocytic antigen (TROG-antigen) from tumor cells to NK cells. TROG-antigen expression decreases CAR-NK cell persistence. In addition, lower TROG-antigen expression favors clinical response^20^.

However, despite the success of early experimental studies, the clinical use of CAR-NK is currently very limited, and the technology must overcome inevitable difficulties and practical challenges. Ideally, reprogramming CAR-NK cells into the memory cells necessary for the long-term surveillance of cancers *in vivo* will be achieved. In addition, the properties of CAR-NK cells must be altered to improve their function in the unfavorable conditions of the TME.

Finally, the design of future immunotherapy strategies must consider how the optimal functioning of NK cells can be achieved within the TME. Thus, further studies investigating the basic science of NK cell biology will also shed light on how to improve NK cell-based immunotherapies.

## References

[1] Siegel RL, Miller KD, Fuchs HE, Jemal A (2022). Cancer statistics, 2022. CA Cancer J Clin.

[2] Zhang C, Yin J, Zheng J, Xiao J, Hu J, Su Y (2021). EZH2 identifies the precursors of human natural killer cells with trained immunity. Cancer Biol Med.

[3] Park MD, Reyes-Torres I, LeBerichel J, Hamon P, LaMarche NM, Hegde S (2023). TREM2 macrophages drive NK cell paucity and dysfunction in lung cancer. Nat Immunol.

[4] Armand P, Lesokhin A, Borrello I, Timmerman J, Gutierrez M, Zhu L (2021). A phase 1b study of dual PD-1 and CTLA-4 or KIR blockade in patients with relapsed/refractory lymphoid malignancies. Leukemia.

[5] Hasim MS, Marotel M, Hodgins JJ, Vulpis E, Makinson OJ, Asif S (2022). When killers become thieves: trogocytosed PD-1 inhibits NK cells in cancer. Sci Adv.

[6] Cong J, Wang X, Zheng X, Wang D, Fu B, Sun R (2018). Dysfunction of natural killer cells by FBP1-induced inhibition of glycolysis during lung cancer progression. Cell Metab.

[7] Zheng X, Qian Y, Fu B, Jiao D, Jiang Y, Chen P (2019). Mitochondrial fragmentation limits NK cell-based tumor immunosurveillance. Nat Immunol.

[8] Orange JS (2008). Formation and function of the lytic NK-cell immunological synapse. Nat Rev Immunol.

[9] Zheng X, Hou Z, Qian Y, Zhang Y, Cui Q, Wang X (2023). Tumors evade immune cytotoxicity by altering the surface topology of NK cells. Nat Immunol.

[10] Knelson EH, Ivanova EV, Tarannum M, Campisi M, Lizotte PH, Booker MA (2022). Activation of tumor-cell STING primes NK-cell therapy. Cancer Immunol Res.

[11] Li F, Liu S (2022). Focusing on NK cells and ADCC: a promising immunotherapy approach in targeted therapy for HER2-positive breast cancer. Front Immunol.

[12] Vyas M, Peigney D, Demehri S (2022). Extracellular matrix-natural killer cell interactome: an uncharted territory in health and disease. Curr Opin Immunol.

[13] Rossi GR, Trindade ES, Souza-Fonseca-Guimaraes F (2020). Tumor microenvironment-associated extracellular matrix components regulate NK cell function. Front Immunol.

[14] Sun X, Wu B, Chiang HC, Deng H, Zhang X, Xiong W (2021). Tumour DDR1 promotes collagen fibre alignment to instigate immune exclusion. Nature.

[15] Wang D, Sun Z, Zhu X, Zheng X, Zhou Y, Lu Y (2022). GARP-mediated active TGF-β1 induces bone marrow NK cell dysfunction in AML patients with early relapse post-allo-HSCT. Blood.

[16] Vago L, Gojo I (2020). Immune escape and immunotherapy of acute myeloid leukemia. J Clin Invest.

[17] Xue W, Zhang M (2021). Updating targets for natural killer/T-cell lymphoma immunotherapy. Cancer Biol Med.

[18] Liu E, Marin D, Banerjee P, Macapinlac HA, Thompson P, Basar R (2020). Use of CAR-transduced natural killer cells in CD19-positive lymphoid tumors. N Engl J Med.

[19] Xie G, Dong H, Liang Y, Ham JD, Rizwan R, Chen J (2020). CAR-NK cells: a promising cellular immunotherapy for cancer. EBioMedicine.

[20] Li Y, Basar R, Wang G, Liu E, Moyes JS, Li L (2022). KIR-based inhibitory CARs overcome CAR-NK cell trogocytosis-mediated fratricide and tumor escape. Nat Med.

